# Circulating amino acid levels and colorectal cancer risk in the European Prospective Investigation into Cancer and Nutrition and UK Biobank cohorts

**DOI:** 10.1186/s12916-023-02739-4

**Published:** 2023-02-28

**Authors:** Joseph A. Rothwell, Jelena Bešević, Niki Dimou, Marie Breeur, Neil Murphy, Mazda Jenab, Roland Wedekind, Vivian Viallon, Pietro Ferrari, David Achaintre, Audrey Gicquiau, Sabina Rinaldi, Augustin Scalbert, Inge Huybrechts, Cornelia Prehn, Jerzy Adamski, Amanda J. Cross, Hector Keun, Marc Chadeau-Hyam, Marie-Christine Boutron-Ruault, Kim Overvad, Christina C. Dahm, Therese Haugdahl Nøst, Torkjel M. Sandanger, Guri Skeie, Raul Zamora-Ros, Kostas K. Tsilidis, Fabian Eichelmann, Matthias B. Schulze, Bethany van Guelpen, Linda Vidman, Maria-José Sánchez, Pilar Amiano, Eva Ardanaz, Karl Smith-Byrne, Ruth Travis, Verena Katzke, Rudolf Kaaks, Jeroen W. G. Derksen, Sandra Colorado-Yohar, Rosario Tumino, Bas Bueno-de-Mesquita, Paolo Vineis, Domenico Palli, Fabrizio Pasanisi, Anne Kirstine Eriksen, Anne Tjønneland, Gianluca Severi, Marc J. Gunter

**Affiliations:** 1 Centre for Epidemiology and Population Health (Inserm U1018), Exposome and Heredity team, Faculté de Médecine, Université Paris-Saclay, UVSQ, Gustave Roussy, F-94805 Villejuif, France; 2 Cancer Epidemiology Unit, Nuffield Department of Population Health, University of Oxford, Oxford, UK; 3 International Agency for Research on Cancer (IARC), 150 cours Albert Thomas, 69008 Lyon, France; 4 Metabolomics and Proteomics Core, Helmholtz Zentrum München, 85764 Neuherberg, Germany; 5 Department of Biochemistry, Yong Loo Lin School of Medicine, National University of Singapore, 8 Medical Drive, Singapore, 117597 Singapore; 6 Institute of Experimental Genetics, Helmholtz Zentrum München, German Research Center for Environmental Health, Ingolstädter Landstraße 1, 85764 Neuherberg, Germany; 7 Institute of Biochemistry, Faculty of Medicine, University of Ljubljana, Vrazov trg 2, 1000 Ljubljana, Slovenia; 8 School of Public Health, Imperial College London, London, UK; 9 Department of Surgery & Cancer, Imperial College London, London, UK; 10 Department of Public Health, Aarhus University, Bartholins Allé 2, DK-8000 Aarhus, Denmark; 11 Faculty of Health Sciences, Department of Community Medicine, UiT the Arctic University of Norway, N-9037 Tromsø, Norway; 12 Unit of Nutrition and Cancer, Cancer Epidemiology Research Programme, Catalan Institute of Oncology (ICO), Bellvitge Biomedical Research Institute (IDIBELL), Barcelona, Spain; 13 Department of Hygiene and Epidemiology, University of Ioannina School of Medicine, Ioannina, Greece; 14 German Center for Diabetes Research (DZD), Munchen-Neuherberg, Germany; 15 Department of Molecular Epidemiology, German Institute of Human Nutrition Potsdam-Rehbruecke, Nuthetal, Germany; 16 Institute of Nutritional Science, University of Potsdam, Potsdam, Germany; 17 Department of Radiation Sciences, Oncology Unit, Umeå University, Umeå, Sweden; 18 Wallenberg Centre for Molecular Medicine, Umeå University, Umeå, Sweden; 19 Escuela Andaluza de Salud Pública (EASP), 18011 Granada, Spain; 20 Instituto de Investigación Biosanitaria ibs. GRANADA, 18012 Granada, Spain; 21 Centro de Investigación Biomédica en Red de Epidemiología y Salud Pública (CIBERESP), 28029 Madrid, Spain; 22 Department of Preventive Medicine and Public Health, University of Granada, 18071 Granada, Spain; 23 Ministry of Health of the Basque Government, Sub Directorate for Public Health and Addictions of Gipuzkoa, San Sebastián, Spain; 24 Biodonostia Health Research Institute, Epidemiology of Chronic and Communicable Diseases Group, San Sebastián, Spain; 25 Spanish Consortium for Research on Epidemiology and Public Health (CIBERESP), Instituto de Salud Carlos III, Madrid, Spain; 26 Navarra Public Health Institute, Leyre 15, 31003 Pamplona, Spain; 27 IdiSNA, Navarra Institute for Health Research, Pamplona, Spain; 28 German Cancer Research Center (DKFZ), Division of Cancer Epidemiology, Heidelberg, Germany; 29 Julius Center for Health Sciences and Primary Care, University Medical Center Utrecht, Utrecht University, Utrecht, The Netherlands; 30 Department of Epidemiology, Murcia Regional Health Council, IMIB-Arrixaca, Murcia, Spain; 31 Research Group on Demography and Health, National Faculty of Public Health, University of Antioquia, Medellín, Colombia; 32 Cancer Registry and Histopathology Department, Provincial Health Authority (ASP), Ragusa, Italy; 33 Department for Determinants of Chronic Diseases (DCD), National Institute for Public Health and the Environment (RIVM), PO Box 1, 3720 BA Bilthoven, The Netherlands; 34 Italian Institute of Technology, Genova, Italy; 35 Cancer Risk Factors and Life-Style Epidemiology Unit, Institute for Cancer Research, Prevention and Clinical Network – ISPRO, Florence, Italy; 36 Dipartimento di Medicina Clinica e Chirurgia, Federico II University, Naples, Italy; 37 Danish Cancer Society Research Center, Diet, Genes and Environment, Strandboulevarden 49, DK-2100 Copenhagen, Denmark; 38 Department of Public Health, University of Copenhagen, Copenhagen, Denmark; 39 Department of Statistics, Computer Science, Applications “G. Parenti” University of Florence, Florence, Italy

**Keywords:** Colorectal cancer, Amino acids, Glutamine, Histidine

## Abstract

**Background:**

Amino acid metabolism is dysregulated in colorectal cancer patients; however, it is not clear whether pre-diagnostic levels of amino acids are associated with subsequent risk of colorectal cancer. We investigated circulating levels of amino acids in relation to colorectal cancer risk in the European Prospective Investigation into Cancer and Nutrition (EPIC) and UK Biobank cohorts.

**Methods:**

Concentrations of 13-21 amino acids were determined in baseline fasting plasma or serum samples in 654 incident colorectal cancer cases and 654 matched controls in EPIC. Amino acids associated with colorectal cancer risk following adjustment for the false discovery rate (FDR) were then tested for associations in the UK Biobank, for which measurements of 9 amino acids were available in 111,323 participants, of which 1221 were incident colorectal cancer cases.

**Results:**

Histidine levels were inversely associated with colorectal cancer risk in EPIC (odds ratio [OR] 0.80 per standard deviation [SD], 95% confidence interval [CI] 0.69–0.92, FDR *P*-value=0.03) and in UK Biobank (HR 0.93 per SD, 95% CI 0.87–0.99, *P*-value=0.03). Glutamine levels were borderline inversely associated with colorectal cancer risk in EPIC (OR 0.85 per SD, 95% CI 0.75–0.97, FDR *P*-value=0.08) and similarly in UK Biobank (HR 0.95, 95% CI 0.89–1.01, *P*=0.09) In both cohorts, associations changed only minimally when cases diagnosed within 2 or 5 years of follow-up were excluded.

**Conclusions:**

Higher circulating levels of histidine were associated with a lower risk of colorectal cancer in two large prospective cohorts. Further research to ascertain the role of histidine metabolism and potentially that of glutamine in colorectal cancer development is warranted.

**Supplementary Information:**

The online version contains supplementary material available at 10.1186/s12916-023-02739-4.

## Background

Colorectal cancer is the third most common cancer globally, with around 1.9 million cases diagnosed in 2020, and the second most common cause of cancer-related death [1]. There is great potential to reduce this burden since most colorectal cancer cases are sporadic [2] and are associated with modifiable risk factors such as body fatness [3], alcohol intake [4], and diet [5]. Colorectal cancer development is also influenced by metabolic factors [6, 7]. For example, insulin and insulin-like growth factors are thought to play causal roles in colorectal tumorigenesis [8], likely through the promotion of cell proliferation and growth signaling pathways [9]. Broad metabolic dysfunction may lead to perturbed small-molecule metabolism, which in turn elicits bioactivity at the level of tissues and organs.

Amino acids are among the most abundant circulating metabolites and serve as building blocks of proteins, precursors of many signaling molecules, and an important energy source via the citric acid cycle. Certain amino acids may also fuel cancer development [10], and marked changes in blood amino acid concentrations have been extensively observed in colorectal cancer patients [11]. For example, levels of amino acids such as glutamine, citrulline, alanine, and histidine have been inversely associated with advancing disease stage [12, 13], while valine and leucine were among the metabolites that distinguished colorectal cancer cases using a discovery-replication strategy [14]. Similarly, the concentrations of several blood amino acids distinguished early-stage colorectal cancer cases from controls in Japanese patients, most notably aspartic acid [15], as well as ornithine and lysine [16]. Glutamine was a notable discriminant in patients newly diagnosed with colorectal cancer compared to controls in a Chinese hospital-based study [17]. Overall, amino acid levels were generally inversely associated with prevalent colorectal neoplasia, suggesting a depletion of serological concentrations in cases compared to healthy individuals. Amino acid profiling could therefore potentially help identify early-stage disease [18], as well as providing insights into mechanisms of carcinogenesis.

Despite these observations, few prospective studies have been conducted to test the hypothesis that pre-diagnostic amino acid concentrations are associated with colorectal cancer risk. Two such studies of nested case-control design that analyzed pre-diagnostic serum or plasma by untargeted metabolomics found limited dysregulation of lipophilic metabolites only [19, 20], while in a case-control study nested in the European Prospective Investigation into Cancer and Nutrition (EPIC) cohort that measured tryptophan and serotonin levels, tryptophan was inversely associated with colon cancer [21]. The aim of the current study was thus to test these associations in a larger and more comprehensive analysis. We first employed the EPIC nested case-control study as a discovery cohort, which measured between 13 and 21 amino acids in fasting plasma or serum in relation to colorectal cancer. In a replication step, we tested those amino acids associated with colorectal cancer risk in EPIC in the UK Biobank cohort, in which 9 overlapping compounds had been measured in over 111,000 participants. Together, the two cohorts allow for the largest and most detailed investigation of circulating amino acids and colorectal cancer risk performed to date.

## Methods

### The EPIC cohort

The EPIC cohort includes over 520,000 individuals who were recruited between 1992 and 2000 from 23 study centers across 10 European countries (Denmark, France, Germany, Greece, Italy, Norway, Spain, Sweden, the Netherlands, and the UK). Participants were 35-70 years of age at recruitment, and approximately 70% of the cohort are women. The study design has been previously described [22, 23]. In brief, extensive questionnaire data on dietary and lifestyle variables were collected at baseline, and approximately 75% of individuals provided non-fasting blood samples.

Incident cases of colorectal cancer were identified through record linkage with regional cancer registries or via a combination of methods, such as the use of health insurance records, contacts with cancer and pathology registries, and active follow-up through participants and their next of kin. Colorectal cancer was defined using the tenth edition of the International Classification of Disease (ICD-10) and the second edition of the International Classification of Disease for Oncology (ICD-O-2). Proximal colon cancers included those found within the cecum, ascending colon, hepatic flexure, transverse colon, and splenic flexure (C18.0 and C18.2–18.5). Distal colon cancers included those found within the descending (C18.6) and sigmoid (C18.7) colon. Overlapping (C18.8) and unspecified (C18.9) lesions of the colon were classed as colon cancers only. Cancer of the rectum included cancers occurring at the recto-sigmoid junction (C19) and rectum (C20).

The current study employed a fasted subset of EPIC data, obtained from two separate metabolomics studies on colorectal cancer, as a discovery cohort. Samples were analyzed using the Biocrates AbsoluteIDQ^TM^ p180 kit (467 cases and 467 matched controls) and the p150 kit (1141 cases and 1141 controls). Combining these studies and then excluding non-fasting participants resulted in a final combined sample of 654 fasted cases and 654 controls, of which 354 case-control pairs were analyzed using the p180 kit. Controls were selected using incidence density sampling from all cohort members who were alive and free of cancer (except non-melanoma skin cancer) at the time of diagnosis of the colorectal cancer cases. Controls were matched to cases on age at recruitment (within 6 months), sex, study center, follow-up time since blood collection, time of day at blood collection (within 4 h), and fasting status. Women were further matched on menopausal status (pre-, peri-, and post-menopausal) and, in pre-menopausal women, phase of menstrual cycle at blood collection. Approval for the study was obtained from the International Agency for Research on Cancer (IARC) and local center review boards. All participants provided written informed consent.

### The UK Biobank cohort

The UK Biobank aims to investigate the genetic, lifestyle, and environmental causes of a range of diseases [24]. Between 2006 and 2010, 502,656 adults aged between 40 and 69 years (229,182 men and 273,474 women) who were registered with the UK National Health Service were recruited at 22 study assessment centers. Ethical approval was obtained from the North West Multicentre Research Ethics Committee, the National Information Governance Board for Health and Social Care in England and Wales, and the Community Health Index Advisory Group in Scotland. All participants provided written informed consent. The present study was undertaken under application number 25897.

During the baseline recruitment visit, participants completed a self-administered questionnaire on socio-demographics (including age, sex, education, and Townsend deprivation score), health and medical history, lifestyle exposures (including smoking habits, dietary intakes, and alcohol consumption), early life exposures, and medication use. Physical measurements were taken, including weight, height, and waist circumference. Colorectal cancer cases were defined using the 10th Revision of the International Classification of Diseases (ICD-10). Colorectal cancers comprised those of the proximal colon (C18.0 and C18.2–18.5), distal colon (C18.6–C18.7), overlapping and unspecified lesions of the colon (C18.8–C18.9), and rectal cancers (C19–C20), as described above.

**Fig. 1:**
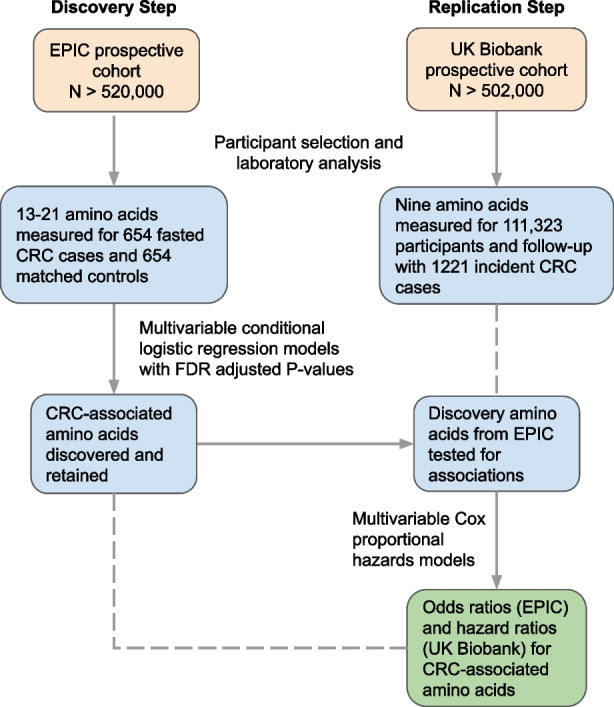
Flow chart showing discovery and replication study design. CRC, colorectal cancer; EPIC, European Prospective Investigation into Nutrition and Cancer

Blood samples, with data on time since last meal, were collected from all participants at recruitment and additionally from around 20,000 participants who attended a repeat assessment visit between 2012 and 2013. The current study included all participants for whom metabolite profiling had been performed at the time of the study, and thus had available amino acid measurements. From our supplied dataset that contained observations for 502,524 participants, exclusions were made for voluntary withdrawal from the study (*n* = 36) and prevalent cancer at recruitment (*n* = 27,240). Of the remainder, plasma amino acid measurements were available for 111,323 participants, and these were included as the replication cohort (Fig. 1).

### Laboratory methods

In EPIC, targeted metabolomics profiling was performed at the International Agency for Research on Cancer (Biocrates AbsoluteIDQ^TM^ p180 kit) and the Helmholtz Centre in Munich (Biocrates AbsoluteIDQ^TM^ p150 kit). The samples were prepared as per the Biocrates kit instructions [25, 26]. Assay preparation steps were carried out on 96 well plates and a volume of 10 μL plasma was prepared. The p150 kit allows the quantification of up to 13 amino acids and the p180 kit up to 21 amino acids (Additional file 1: Supplemental Methods) [25, 27]. Liquid chromatography–mass spectrometry (LC-MS) was used to quantify the levels of the amino acids in accordance with the kit manufacturer’s instructions. All 21 amino acids included were fully quantified in μmol/L. The amino acids quantified were arginine, glutamine, glycine, histidine, methionine, ornithine, phenylalanine, proline, serine, threonine, tryptophan, tyrosine, and valine (p150 and p180 kits); and alanine, asparagine, aspartate, citrulline, glutamate, isoleucine, leucine, and lysine (p180 kit only). See Additional file 1: Supplemental Methods for full details of sample preparation. Coefficients of variation for amino acids are given in Table S1.

Analysis of plasma from around 118,000 participants of the UK Biobank was performed using nucleic magnetic resonance (NMR) spectroscopy on the Nightingale metabolic biomarker platform (Nightingale Health Ltd, Finland), which comprises 249 metabolic measures, among which are concentrations of 9 amino acids. In brief, stored plasma samples prepared in 96-well plates were thawed, mixed gently, and centrifuged for 3 min at 3400 g to remove the precipitate. Aliquots of each sample were mixed with phosphate buffer, loaded onto a cooled sample changer, and analyzed by NMR spectroscopy. Metabolic biomarkers were identified and quantified from two separate spectra, a pre-saturated proton NMR spectrum, and a T2-relaxation-filtered spectrum. Six identical Bruker AVANCE IIIHD instruments were employed in parallel. The amino acids quantified were alanine, glutamine, glycine, histidine, isoleucine, leucine, valine, phenylalanine, and tyrosine. See Additional file 1: Supplemental Methods for further details.

### Statistical analysis

Pearson correlations between non-log transformed amino acid concentrations were first calculated in the 654 EPIC fasted controls only and in UK Biobank participants with a fasted time of >4 h (*n* = 56,688). Hierarchical clustering of concentration profiles using Ward’s method was used to visualize and identify notable clusters of correlated metabolites.

#### *Analysis of discovery cohort*

In EPIC, case-control status was modeled using conditional logistic regression and odds ratios (OR) and 95% confidence intervals (CI) estimated for each amino acid. Models were adjusted for an a priori determined set of potential confounders comprising smoking status (never, former, current, unknown), alcohol drinking history (never, former, current, lifetime, unknown), Cambridge physical activity index (inactive, moderately inactive, moderately active, active, unknown) and body mass index (BMI; <25, 25–30, and >30kg/m^2^), all at baseline. The false discovery rate (FDR) procedure was used to adjust *P*-values and an FDR *P-*value threshold of 0.05 was used for statistical significance. Continuous models per SD concentration and categorical models by quartile were fit for each amino acid. For the categorical models, inner quartile cut points were determined by the metabolite concentrations among control participants. To test for trends across categories, quartile medians were additionally modeled as continuous variables.

#### *Analysis of replication cohort*

Amino acids that were significantly associated with colorectal cancer per SD concentration in EPIC were carried forward for testing in the UK Biobank cohort. Here, time to colorectal cancer diagnosis was modeled using Cox proportional hazards regression and hazard ratios (HR) and 95% CI estimated for each amino acid. Time at study entry was age at recruitment, while exit time was age at incident cancer diagnosis, death, or the last date at which follow-up was considered complete. Multivariable models were kept as similar as possible to those fit in EPIC and were adjusted for BMI category (<25, 25–30, >30 kg/m^2^), total physical activity (<10, 10–20, 20–40, 40–60, >60 metabolic equivalent of task [MET] h/week), alcohol consumption frequency (never, special occasions only, 1–3 times/month, 1–2 times per week, 3–4 times/week, daily or almost daily, unknown/prefer not to answer), smoking status (smoker, former smoker, never smoker), time since last meal (hours), and family history of colorectal cancer (yes/no). Stratification variables were age at recruitment in 5-year intervals, Townsend deprivation index quintiles, and assessment center region. A raw P-value threshold of 0.05 was used for statistical significance.

#### *Stratified and sensitivity analyses*

The above analysis was repeated but excluding individuals diagnosed within the first 2, 5, and 10 years of the study in EPIC, and within the first 2 and 5 years in the UK Biobank. Sex-stratified models were also performed for all amino acids measured in both cohorts and, in UK Biobank, amino acid models were conducted for colon and rectal subsites separately. Heterogeneity by sex and by tumor subsite was tested for by fitting models with and without interaction terms and comparing these by likelihood ratio test. As sensitivity analyses, models for glutamine and histidine only were repeated additionally adjusting for major sources of animal proteins (red and processed meat, poultry, fish, and dairy product intake), and amino acid models were repeated in EPIC only using non-fasted participants as well as fasted participants.

Analyses were conducted either in the R open-source statistical programming language (version 3.6.3 on the RStudio environment) or STATA version 16.1 (StataCorp Inc).

## Results

**Fig. 2:**
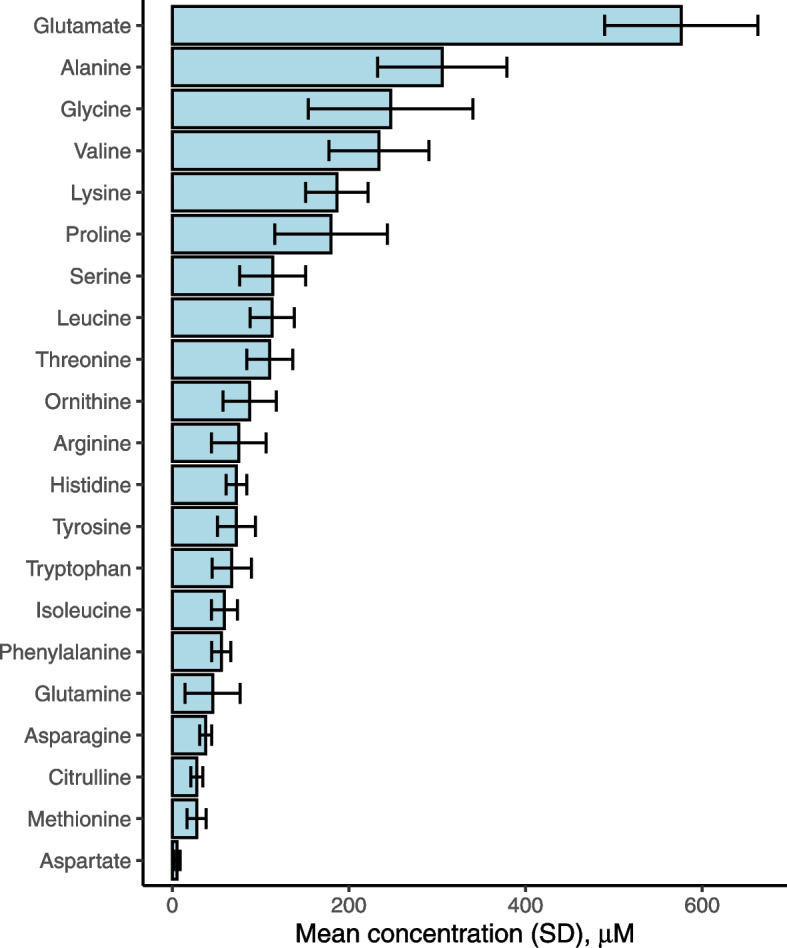
Blood concentrations of amino acids as determined in fasted EPIC participants on the p150 or p180 Biocrates platform. Based on 654 and 354 cancer-free controls for p150 and p180 platforms, respectively

**Fig. 3:**
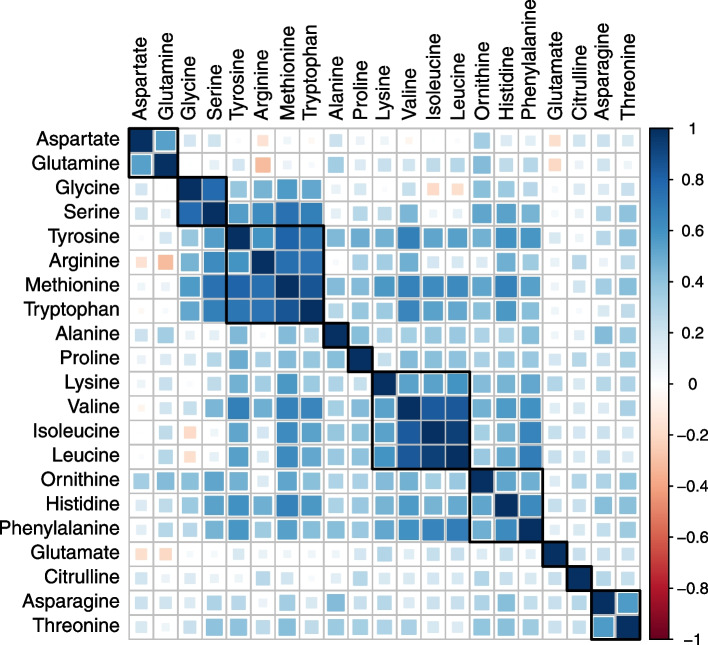
Fasting amino acid concentrations and their intercorrelations in EPIC cancer-free controls. Compounds are ordered by the hierarchical cluster as determined by Ward’s method. Squares represent groups of highly correlated compounds

A median follow-up of 14.4 years was observed for the 654 colorectal cancer cases and 654 controls in EPIC while, during a median follow-up of 10.7 years in the UK Biobank, 1221 incident cases of colorectal cancer occurred among the 111,323 participants with available amino acid measurements. The EPIC and UK Biobank populations were of similar ages at baseline and at colorectal cancer diagnosis although, in EPIC, most participants (77.4%) were from Italian or Spanish centers. Full baseline characteristics are shown in Table 1. Glutamine, alanine, and glycine were at the highest circulating concentrations overall, as quantified in EPIC (Fig. 2). Fasting concentrations of amino acids in cancer-free participants were almost always positively correlated, with the following correlated clusters noted in EPIC: glycine and serine; arginine, methionine, and tryptophan; valine, isoleucine, and leucine; and histidine and phenylalanine (Fig. 3). In the UK Biobank, valine, isoleucine, and leucine concentrations (branched-chain amino acids) were strongly intercorrelated.

**Table 1:** Baseline characteristics of participants, by cohort

	EPIC nested case-control study (*n* = 654 cases and 654 controls)		UK Biobank Prospective cohort (*n* = 111,323 of which 1221 incident colorectal cancers)	
	Controls	Cases	Non-cases	Cases
**Sex**				
Male	288 (44.0)	288 (44.0)	59,295 (53.9)	513 (42.0)
Female	366 (56.0)	366 (56.0)	50,807 (46.1)	708 (58.0)
**Country**				
France	29 (4.4)	29 (4.4)	-	
Italy	336 (41.4)	336 (41.4)	-	
Spain	235 (36.0)	235 (36.0)	-	
UK	23 (3.5)	23 (3.5)	110,102	1221
Netherlands	2 (0.3)	2 (0.3)	-	
Germany	27 (4.1)	27 (4.1)	-	
Denmark	2 (0.3)	2 (0.3)	-	
**Age at blood collection (years)**				
Mean	54.7 ± 7.3	54.8 ± 7.3	56.3 ± 8.1	61.0 ± 6.6
**Follow-up time to diagnosis (years)**				
Mean	-	8.9 ± 4.5	-	9.7 ± 2.0
**BMI (kg/m**^**2**^**)**				
Mean (SD)	26.7 ± 3.8	27.5 ± 4.4	27.4 ± 4.8	28.0 ± 4.8
**Waist circumference (cm)**				
Mean (SD)	88.6 ± 12.0	90.7 ± 13.6	90.3 ± 13.4	94.0 ± 14.1
**Height (cm)**				
Mean (SD)	163.1 ± 9.0	163.7 ± 9.0	168.5 ± 9.3	169. 9 ± 9.1
**Smoking status**				
Non or former smoker	334 (51.1)	308 (47.1)	60,105 (54.6)	549 (45.0)
Never smoker	176 (26.9)	171 (26.1)	37,735 (34.3)	547 (44.8)
Smoker	141 (21.6)	170 (26.0)	11,694 (10.6)	123 (10.1)
Unknown	3 (0.5)	5 (0.8)	568 (0.5)	2 (0.2)
**Alcohol intake status**				
Never drinker	62 (9.5)	69 (10.6)	4837 (4.3)	4837 (4.3)
Former drinker	54 (8.3)	47 (7.2)	3965 (3.6)	3965 (3.6)
Drinker at recruitment (current)	509 (77.8)	504 (77.1)	102,251 (91.8)	102,251 (91.8)
Unknown	29 (4.4)	34 (5.2)	274 (0.2)	274 (0.2)
**Physical activity status**				
Inactive	214 (32.7)	234 (35.8)	31,763 (28.8%)	350 (28.7)
Moderately inactive	255 (39.0)	252 (38.5)	21,532 (19.6%)	246 (20.1)
Moderately active	105 (16.1)	105 (16.1)	35,798 (32.5%)	375 (30.7)
Active	79 (12.1)	62 (9.5)	17,086 (15.5%)	214 (17.5)
Missing	1 (0.2)	1 (0.2)	3923 (3.6%)	36 (2.9)
**Highest educational level**				
None	95 (14.5)	98 (15.2)	0	0
Primary school completed	274 (42.0)	261 (40.6)	0	0
Technical/professional school	84 (12.9)	73 (11.4)	31,067 (28.2)	332 (27.2)
Secondary school	104 (15.9)	125 (19.4)	46,786 (42.0)	468 (38.3)
Higher education/university	90 (13.8)	79 (12.3)	12,509 (11.4)	141 (11.5)
Not specified	6 (0.9)	7 (1.1)	19,740 (17.7)	280 (22.9)
**Oral contraceptive use in women**				
Ever	5 (1.4)	5 (1.4)	11,033 (10.0)	119 (9.7)
Never	360 (98.4)	361 (98.6)	47,994 (43.6)	391 (32.0)
Unknown	1 (0.0)	0 (0.0)	51,075 (46.4)	711 (58.2)
**Oral hormone therapy use in women**				
Ever	31 (8.5)	37 (10.1)	36,987 (61.8)	36,716 (33.3)
Never	334 (91.3)	326 (89.1)	22,515 (37.6)	22,276 (20.2)
Unknown	1 (0.0)	3 (0.01)	307 (0.0)	303 (0.3)

Means and SD or frequency and percentage are shown unless stated otherwise

*SD*, standard deviation; *BMI*, body mass index

### Associations of pre-diagnostic amino acid concentrations with colorectal cancer risk

In the EPIC discovery phase, histidine concentrations were inversely associated with colorectal cancer risk (OR 0.80 per SD concentration, 95% CI 0.69–0.92, FDR *P*-value = 0.03) (Table 2). A statistically significant trend was also observed by quartile of histidine concentration (*P*-trend = 0.002). Lysine was also inversely associated with colorectal cancer risk (OR 0.78 per SD concentration, 95% CI 0.66–0.93, FDR *P*-value = 0.05), and glutamine was borderline inversely associated with risk (OR 0.85 per SD concentration, 95% CI 0.75–0.97, FDR *P*-value = 0.08). For both lysine and glutamine, individuals in Q4 of concentrations had a lower risk compared to those in Q1, with an apparent decreasing trend across quartiles (*P*-trend for both amino acids = 0.01).

**Table 2:** Associations between concentrations of 21 plasma or serum amino acids and colorectal cancer risk in the EPIC nested case-control discovery and UK Biobank replication cohorts

Amino acid (by decreasing blood concentration)	Colorectal cancer cases ^a^	OR/HR per SD concentration (95% CI) ^b^	FDR *P*-value	OR/HR (95% CI) for Q2 ^bc^	OR/HR (95% CI) for Q3 ^bc^	OR/HR (95% CI) for Q4 ^bc^	*P*-trend ^d^
**Discovery cohort (EPIC nested case-control study)**							
Glutamine	654	**0.85 (0.75–0.97)**	0.08	0.84 (0.61–1.16)	0.72 (0.52–1.01)	**0.65 (0.46–0.92)**	0.01
Alanine	354	1.04 (0.89–1.22)	0.75	1.04 (0.68–1.64)	1.06 (0.68–1.64)	1.24 (0.79–1.95)	0.47
Glycine	654	0.89 (0.75–1.05)	0.45	0.99 (0.70–1.40)	0.82 (0.54–1.24)	0.73 (0.46–1.16)	0.08
Valine	654	0.92 (0.78–1.08)	0.47	0.98 (0.69–1.38)	0.76 (0.52–1.11)	0.91 (0.58–1.41)	0.45
Lysine	354	**0.78 (0.66–0.93)**	0.05	0.82 (0.53–1.28)	0.83 (0.53–1.31)	**0.51 (0.31–0.83)**	0.008
Proline	654	1.06 (0.93–1.20)	0.48	1.02 (0.74–1.42)	1.15 (0.82–1.62)	1.05 (0.72–1.52)	0.73
Serine	654	0.89 (0.73–1.08)	0.45	0.83 (0.60–1.16)	0.73 (0.49–1.09)	0.61 (0.37–1.01)	0.05
Leucine	354	**0.79 (0.64–0.98)**	0.15	0.99 (0.64–1.54)	0.72 (0.44–1.21)	0.75 (0.44–1.29)	0.20
Threonine	654	0.96 (0.85–1.09)	0.60	1.14 (0.83–1.56)	0.85 (0.61–1.19)	0.97 (0.68–1.37)	0.50
Ornithine	654	0.88 (0.69–1.13)	0.47	0.90 (0.63–1.28)	0.63 (0.35–1.15)	0.65 (0.33–1.28)	0.73
Arginine	654	1.02 (0.83–1.24)	0.87	1.18 (0.85–1.65)	0.93 (0.59–1.45)	0.98 (0.57–1.68)	0.91
Tyrosine	654	0.90 (0.76–1.06)	0.45	0.92 (0.67–1.27)	0.89 (0.61–1.28)	0.84 (0.54–1.32)	0.41
Histidine	654	**0.80 (0.69–0.92)**	0.03	0.79 (0.57–1.09)	**0.59 (0.41–0.84)**	**0.61 (0.42–0.89)**	0.005
Tryptophan	654	0.85 (0.64–1.12)	0.45	**0.62 (0.43–0.88)**	**0.53 (0.29–0.96)**	0.55 (0.27–1.13)	0.07
Isoleucine	354	0.92 (0.75–1.14)	0.64	0.93 (0.59–1.46)	0.75 (0.45–1.23)	0.85 (0.49–1.47)	0.39
Phenylalanine	354	0.89 (0.77–1.03)	0.45	1.24 (0.89–1.74)	0.97 (0.68–1.39)	0.86 (0.58–1.28)	0.13
Glutamate	354	1.10 (0.85–1.43)	0.64	**1.71 (1.04–2.81)**	**2.02 (1.16–3.52)**	1.82 (0.96–3.45)	0.19
Asparagine	354	1.08 (0.91–1.28)	0.58	1.10 (0.70–1.72)	1.40 (0.89–2.20)	0.93 (0.57–1.53)	0.91
Methionine	654	0.88 (0.69–1.13)	0.47	0.90 (0.63–1.28)	0.63 (0.35–1.15)	0.65 (0.33–1.28)	0.20
Citrulline	354	1.15 (0.97–1.37)	0.40	**0.60 (0.37–0.97)**	1.06 (0.69–1.64)	1.11 (0.69–1.78)	0.36
Aspartate ^e^	354	0.99 (0.81–1.21)	0.91	-	-	-	-
**Replication cohort (UK Biobank cohort)**							
Glutamine	1221	0.95 (0.89–1.01)	0.09	0.89 (0.76–1.05)	0.99 (0.84–1.16)	**0.85 (0.72–1.00)**	0.12
Histidine	1221	**0.93 (0.87–0.99)**	0.03	1.01 (0.86–1.17)	**0.76 (0.65–0.90)**	0.86 (0.73–1.01)	0.008

*OR*, odds ratio; *HR*, hazard ratio; *FDR*, false discovery rate; *Q*, quartile. Estimates for which 95% CI do not include 1 are given in bold text

^a^ In EPIC, amino acids measured using the Biocrates AbsoluteIDQ^TM^ p180 kit were measured in 354 cases and 354 controls only

^b^ Multivariable models were adjusted for smoking status (never, former, and current smoker), alcohol use (never, former, only at recruitment, and lifetime drinker), physical activity at recruitment (inactive, moderately inactive, moderately active, active) and body mass index (<25, 25–30, and >30 kg/m^2^). In UK Biobank, categories differed slightly for total physical activity (<10, 10–20, 20–40, 40–60, >60 metabolic equivalent of task [MET] h/week), alcohol consumption frequency (never, special occasions only, 1–3 times/month, 1–2 times per week, 3–4 times/week, daily or almost daily, unknown/prefer not to answer), and were also adjusted for family history of colorectal cancer (yes/no) and time since last meal (hours)

^c^ Q1 of amino acid concentrations was considered the referent group. Inner quartile cut points were determined by metabolite concentrations in controls only

^d^ Calculated by the replacement of continuous data by the median values of concentration quartiles in mode

^e^ The categorical analysis was not performed due to a high proportion of missing values

Histidine and glutamine, but not lysine, were among the nine amino acids measured in UK Biobank and were thus carried forward to the replication stage. Histidine was also significantly inversely associated with colorectal risk in UK Biobank (HR 0.93 per SD concentration, 95% CI 0.87–0.99, *P*-value = 0.03), with a significantly decreasing trend across quartiles of concentration. Glutamine was again borderline inversely associated with risk on a continuous scale (HR 0.95 per SD, 95% CI 0.89–1.01 respectively, *P*-value = 0.09), and individuals in Q4 of concentrations were at a lower risk of colorectal cancer than those in Q1 (HR 0.85, 95% CI 0.72–1.00).

### Analysis by follow-up time

In EPIC, ORs for histidine and glutamine did not appreciably change when cases diagnosed within 2, 5, or 10 years were excluded (OR 0.82 per SD concentration, 95% CI 0.67–1.01 and OR 0.82, 95% CI 0.68–0.99 respectively for the two amino acids, exclusion of the first 10 years of follow-up) (Table 3). Similarly, minor changes in HR were observed for the exclusion of 2 and 5-year periods of follow-up for these amino acids in UK Biobank.

**Table 3:** Associations between concentrations of serum and plasma amino acids and colorectal cancer risk in the EPIC nested case-control discovery and UK Biobank replication cohorts by follow-up time to diagnosis, where available

Amino acid ^a^		OR/HR (95% CI) per SD concentration by follow-up time ^b^		
	Full follow-up	>2 years	>5 years	>10 years
**EPIC nested case-control study**	*n* = 654 cases, 654 controls	*n* = 601 cases, 601 controls	*n* = 505 cases, 505 controls	*n* = 289 cases, 289 controls
Glutamine	**0.85 (0.75–0.97)**	**0.85 (0.75–0.97)**	**0.83 (0.72–0.96)**	**0.82 (0.68–0.99)**
Glycine	0.89 (0.75–1.05)	0.92 (0.77–1.11)	0.90 (0.73–1.10)	0.92 (0.70–1.21)
Valine	0.92 (0.78–1.08)	0.91 (0.77–1.08)	0.93 (0.77–1.12)	1.04 (0.82–1.33)
Proline	1.06 (0.93–1.20)	1.09 (0.95–1.24)	1.08 (0.94–1.25)	0.96 (0.80–1.15)
Serine	0.89 (0.73–1.08)	0.91 (0.74–1.11)	0.85 (0.68–1.05)	0.81 (0.61–1.06)
Threonine	0.96 (0.85–1.09)	0.97 (0.85–1.10)	0.93 (0.81–1.07)	0.87 (0.72–1.04)
Ornithine	0.88 (0.69–1.13)	0.98 (0.82–1.17)	0.98 (0.80–1.20)	1.03 (0.78–1.36)
Arginine	1.02 (0.83–1.24)	1.04 (0.84–1.28)	1.07 (0.85–1.35)	1.02 (0.77–1.36)
Tyrosine	0.90 (0.76–1.06)	0.89 (0.75–1.06)	0.87 (0.72–1.05)	0.90 (0.71–1.12)
Histidine	**0.80 (0.69–0.92)**	**0.81 (0.69–0.94)**	**0.81 (0.69–0.96)**	0.82 (0.67–1.01)
Tryptophan	0.85 (0.64–1.12)	0.80 (0.59–1.08)	0.77 (0.55–1.06)	0.77 (0.52–1.12)
Phenylalanine	0.89 (0.77–1.03)	0.87 (0.75–1.02)	0.84 (0.71–1.00)	0.83 (0.67–1.03)
Methionine	0.88 (0.69–1.13)	0.86 (0.67–1.12)	0.84 (0.64–1.11)	0.89 (0.65–1.22)
**UK Biobank cohort (*N*** **= 111,323)**	*n* = 1221 cases	*n* = 1042 cases	*n* = 696 cases	
Glutamine	0.95 (0.89–1.01)	0.94 (0.88–1.01)	0.98 (0.90–1.06)	
Histidine	**0.93 (0.87–0.99)**	0.95 (0.88–1.01)	0.94 (0.87–1.03)	

*OR*, odds ratio; *Q*, quartile. Estimates for which 95% CI do not include 1 are given in bold text

^a^ In decreasing order of measured blood concentration. Only amino acids measured in all participants in EPIC were included

^b^ Multivariable models were adjusted for smoking status (never, former, and current smoker), alcohol use (never, former, only at recruitment, and lifetime drinker), physical activity at recruitment (inactive, moderately inactive, moderately active, active) and body mass index (<25, 25–30, and >30 kg/m^2^). ). In UK Biobank, categories differed slightly for total physical activity (<10, 10–20, 20–40, 40–60, >60 metabolic equivalent of task [MET] h/week), alcohol consumption frequency (never, special occasions only, 1–3 times/month, 1–2 times per week, 3–4 times/week, daily or almost daily, unknown/prefer not to answer), and were also adjusted for family history of colorectal cancer (yes/no) and time since last meal (hours)

### Stratified and sensitivity analysis

In EPIC, most available colorectal samples were for cancers of the colon (625/654) and estimates for colon cancer mirrored those of colorectal cancer (Additional file 1: Table S2). Nevertheless, in the UK Biobank where 31.7% of colorectal cases (388/1221) were rectal cancers, HRs for colorectal and colon cancers were also similar. Here, glutamine concentrations were similarly associated with risk of colorectal cancer and colon cancer only (HR 0.92 per SD concentration, 95% CI 0.85–0.99), while no association was observed for rectal cancer (HR 1.02 per SD, 95% CI 0.91-1.13). Heterogeneity between colon and rectal tumor subsites approached but did not reach statistical significance (*P*=0.13). As regards histidine, hazard ratios were similar for colon cancer (HR 0.95, 95% CI 0.88-1.02), rectal cancer (HR 0.89, 95% CI 0.80–0.99), and colorectal cancer overall (HR 0.93, 95% CI 0.87-0.99). Inverse associations for amino acids were more pronounced in men than in women (Additional file 1: Table S3), and heterogeneity by sex was observed for histidine in UK Biobank (*P*-heterogeneity = 0.02). Heterogeneity by sex was not observed for any other amino acid measured in UK Biobank or for any amino acid measured in EPIC.

For the EPIC and UK Biobank participants included in the main study, adjustment for major sources of amino acid intake (red and processed meat, poultry, fish, eggs, and dairy products) did not change associations between circulating amino acids and colorectal cancer risk (Additional file 1: Table S4). Likewise, in sensitivity analyses including non-fasting as well as fasting participants in EPIC, associations did not change appreciably (Additional file 1: Table S5).

## Discussion

In this analysis of pre-diagnostic circulating amino acid levels and colorectal cancer risk, histidine was found to be robustly inversely associated and glutamine borderline inversely associated with colorectal cancer risk via a discovery-replication strategy in two large prospective cohorts. In addition, odds ratios and hazards ratios for these amino acids were attenuated minimally by the exclusion of cases diagnosed within 10 years of follow-up. This study provides strong evidence that lower levels of histidine, and possibly glutamine, are associated with subsequent risk of colorectal cancer, even up to 10 years before a colorectal cancer diagnosis.

Circulating levels of several amino acids have previously been found to be inversely associated with colorectal neoplasia, but in studies of cross-sectional design only. Glutamine, for example, was one of several amino acids found to be lower in colorectal cancer patients compared to healthy controls [28], while histidine was lower among stage IV colorectal cancer cases than stage I cases [12] and even inversely correlated with tumor stage [29]. Untargeted metabolomics studies using discovery and validation cohorts demonstrated leucine and the dipeptide glutamine-leucine to be among those metabolites that distinguished cases from controls [14, 30]. Nevertheless, few studies have analyzed pre-diagnostic samples to investigate whether amino acid dysregulation precedes tumorigenesis. Two other prospective case-control studies on colorectal cancer with some amino acid measurements also found no significant associations [19, 31]. Our study is therefore the first to observe inverse associations of amino acid levels with colorectal cancer risk in a prospective setting and in independent studies. Although the above evidence suggests that tumor energy requirements give rise to the depletion of circulating glutamine and histidine, the levels of these amino acids among colorectal cancer cases in our prospective cohorts were associated with colorectal cancer risk at least 10 years prior to a colorectal cancer diagnosis, suggesting that alterations in the metabolism of these compounds may either reflect etiological pathways associated with the development of disease or metabolic changes linked to early events in colorectal tumorigenesis.

The most pronounced finding of the current study was a robust inverse association between circulating histidine and colorectal cancer risk in both EPIC and UK Biobank. Histidine, an essential amino acid derived from the diet, is converted by histidine decarboxylase to the biogenic amine histamine [32], a signaling molecule that mediates an acute inflammatory response by binding to specific receptors [33]. Histidine decarboxylase activity may be upregulated in tumor cells and is thought to accelerate cell proliferation and angiogenesis [34]. For instance, the enzyme was found to be more active in colon cancer cells, particularly metastatic tumor cells, than normal colonic cells [34]. Given that an inverse association between histidine and colorectal cancer risk was apparent as long as 10 years before diagnosis, perturbations to specific etiologic pathways may be hypothesized. Prior evidence suggests that higher histidine concentrations mitigate metabolic dysregulation; for example, dietary supplementation with histidine was found to improve insulin sensitivity, possibly via the suppression of pro-inflammatory cytokine expression, in women with metabolic syndrome [35]. These findings suggested that histidine may even hold potential as a therapeutic agent against metabolic disease. However, levels of histidine have also been found to be positively associated with breast cancer [36]. Therefore, additional laboratory studies are needed to elucidate the potential role of this amino acid in carcinogenesis.

Glutamine was borderline inversely associated with colorectal cancer risk in both cohorts. Glutamine is among the most abundant small-molecule metabolites in circulation and plays a central role in amino acid metabolism. It is used by proliferating cancer cells as an energy source [37] and is likely an important substrate throughout colorectal tumorigenesis [38]. The reasons for lower circulating glutamine in individuals who went on to develop colorectal cancer compared to those who remained cancer-free are uncertain. Firstly, given the slow development of colorectal cancer, lowered glutamine may reflect the undetected presence of polyps or early cancerous lesions in cases at baseline [28]. Secondly, regardless of the presence of such lesions and even controlling for major risk factors, abnormally low glutamine levels may reflect cancer-promoting metabolism. For instance, as well as being directly related to the tumor stage, glutamine levels have been inversely associated with serum C-reactive protein and inflammatory cytokines [13, 39]. Also, lowered glutamine and the glutamine-glutamate ratio was reported to be associated with incident type II diabetes [40], an established risk factor for colorectal cancer [41]. Lowered glutamine may represent dysregulation of the glutamine-glutamate axis. Although our study measured glutamate in a subset of EPIC cases and controls only, some evidence for a positive association of glutamate with colorectal cancer risk was observed in the categorical analysis. Glutamine concentrations may influence multiple mechanisms related to cancer development which deserve further investigation in experimental models.

The inverse associations observed between amino acid concentrations and colorectal cancer risk may also reflect cancer-promoting dysbiosis of the gut microbiota. Similar to the protection against colorectal cancer afforded by short-chain fatty acids produced from dietary fiber by microbiota via the mitigation of an inflammatory microenvironment [42], certain components of the gut microbiota may act upon amino acids in the lumen to influence inflammation and tumorigenesis [43]^,^[44]. For example, the production of histidine decarboxylase by gut microbes has been suggested to decrease intestinal inflammation via the binding of histamine to the receptor HR2 in the gut lumen [45]. Also, specific components of the microbiota are also believed to mediate the relationship between branched-chain amino acids and insulin resistance [46]. It is therefore plausible that the associations of histidine and glutamine with colorectal cancer risk in the current study may reflect variability in gut microbial activity and its interaction with host metabolism. Further mechanistic research is needed to investigate links between histidine and glutamine metabolism, the gut microbiota and colorectal tumorigenesis.

The main strengths of this study include the prospective design, the use of large-scale cohorts with extensive participant data, and robust amino acid measurements in participants of well-characterized fasting status. We excluded non-fasted participants from the outset in the EPIC discovery cohort to minimize the effects of recent dietary intake upon amino acid levels which could have complicated interpretation of the results. As an additional safeguard against this bias, we performed sensitivity analyses for fasting status in EPIC and for major dietary sources of amino acids in both cohorts, which did not appreciably attenuate risk estimates. This is consistent with a recent study in EPIC that found weak or no correlations between amino acid intake and their blood concentrations [47].

In terms of limitations, only 9 of the 21 amino acids were measured in all EPIC and UK Biobank samples, while only 13 were measured all EPIC samples, with limited statistical power for the remainder. It is plausible that levels of amino acids other than glutamine and histidine are associated with colorectal cancer and we note that HR or OR point estimates were lower than 1 for most compounds in both cohorts. With greater statistical power, particularly in UK Biobank, other amino acids would likely have been found inversely associated with colorectal cancer risk via the discovery-replication strategy. Also, measurements of amino acids were taken at the study baseline only, and the technical and biological reproducibility of measurement was therefore not accounted for. However, studies calculating intra-class correlation in blood samples suggest that polar metabolites such as amino acids are measured reproducibly, particularly in fasted participants [48]. Statistical power was limited for individual colorectal cancer subsites, particularly rectal cancer. Also, we were not able to consider amino acids in tissue samples, which may better represent the tumor microenvironment and provider deeper insight into the biological implications of our findings.

## Conclusions

Circulating histidine levels were robustly inversely associated with colorectal cancer risk in two independent prospective cohorts with similar, albeit slightly weaker, evidence for glutamine. This knowledge should contribute to a better understanding of the underpinnings of colorectal cancer and metabolism and could potentially support new prevention or early detection strategies. Further research using experimental models to assess potential causality of the identified associations is now needed.

## Supplementary Information

**Additional file 1.** Supplemental methods for laboratory analyses; Table S1, Reported coefficients of variation for 21 amino acids measured in the EPIC and UK Biobank cohorts; Table S2, Associations between 21 plasma or serum amino acids and colorectal, colon and rectal cancer risk in the EPIC and UK Biobank cohorts; Table S3, Associations between 21 plasma or serum amino acids and colorectal cancer risk in the EPIC and UK Biobank cohorts, by sex; Table S4, Associations between amino acids associated with colorectal cancer in either cohort in the main study, additionally adjusted for intakes of major sources of animal protein (red and processed meat, poultry, fish and dairy products) in the EPIC and UK Biobank cohorts; Table S5, Associations between concentrations of 21 plasma or serum amino acids and colorectal cancer risk in fasted participants only and all available participants in the EPIC nested case-control study.

## Data Availability

Data from EPIC are not publicly available, but access requests can be made to the to the EPIC Steering Committee (https://epic.iarc.fr/access/submit_appl_access.php). The UK Biobank is an open access resource. This research was conducted under license from the UK Biobank, application number 25897. Data are available upon application to UK Biobank; see http://ukbiobank.ac.uk/register-apply/.

## Abbreviations

BCAA: Branched-chain amino acid
BMI: Body mass index
CI: Confidence interval
HR: Hazard ratio
EPIC: European Prospective Investigation into Cancer and Nutrition
FDR: False discovery rate
IARC: International Agency for Research on Cancer
ICD: International Classification of Diseases
LC-MS: Liquid chromatography–mass spectrometry
MET: Metabolic equivalent of task
NMR: Nuclear magnetic resonance
OR: Odds ratio

## References

[1] Sung H, Ferlay J, Siegel RL, Laversanne M, Soerjomataram I, Jemal A, Bray F (2021). Global cancer statistics 2020: GLOBOCAN estimates of incidence and mortality worldwide for 36 cancers in 185 countries. CA: Cancer J Clin.

[2] Weitz J, Koch M, Debus J, Höhler T, Galle PR, Büchler MW (2005). Colorectal cancer. Lancet.

[3] Abar L, Vieira AR, Aune D, Sobiecki JG, Vingeliene S, Polemiti E, Stevens C, Greenwood DC, Chan DSM, Schlesinger S (2018). Height and body fatness and colorectal cancer risk: an update of the WCRF-AICR systematic review of published prospective studies. Eur J Nutr.

[4] Park JY, Dahm CC, Keogh RH, Mitrou PN, Cairns BJ, Greenwood DC, Spencer EA, Fentiman IS, Shipley MJ, Brunner EJ (2010). Alcohol intake and risk of colorectal cancer: results from the UK Dietary Cohort Consortium. Br J Cancer.

[5] Tabung FK, Brown LS, Fung TT (2017). Dietary Patterns and Colorectal Cancer Risk: a Review of 17 Years of Evidence (2000-2016). Curr Colorectal Cancer Rep.

[6] Giovannucci E, Michaud D (2007). The role of obesity and related metabolic disturbances in cancers of the colon, prostate, and pancreas. Gastroenterology.

[7] Gunter MJ, Alhomoud S, Arnold M, Brenner H, Burn J, Casey G, Chan AT, Cross AJ, Giovannucci E, Hoover R (2019). Meeting report from the joint IARC–NCI international cancer seminar series: a focus on colorectal cancer. Ann Oncol.

[8] Murphy N, Song M, Papadimitriou N, Carreras-Torres R, Langenberg C, Martin RM (2022). Associations Between Glycemic Traits and Colorectal Cancer: A Mendelian Randomization Analysis. J Natl Cancer Inst..

[9] Giovannucci E, Harlan DM, Archer MC, Bergenstal RM, Gapstur SM, Habel LA, Pollak M, Regensteiner JG, Yee D (2010). Diabetes and Cancer A consensus report. Diabetes Care.

[10] Lieu EL, Nguyen T, Rhyne S, Kim J (2020). Amino acids in cancer. Exp Mol Med.

[11] Goveia J, Pircher A, Conradi L-C, Kalucka J, Lagani V, Dewerchin M, Eelen G, DeBerardinis RJ, Wilson ID, Carmeliet P (2016). Meta-analysis of clinical metabolic profiling studies in cancer: challenges and opportunities. EMBO Mol Med.

[12] Geijsen A, van Roekel EH, van Duijnhoven FJB, Achaintre D, Bachleitner-Hofmann T, Baierl A, Bergmann MM, Boehm J, Bours MJL, Brenner H (2019). Plasma metabolites associated with colorectal cancer stage: Findings from an international consortium. Int J Cancer.

[13] Sirniö P, Väyrynen JP, Klintrup K, Mäkelä J, Karhu T, Herzig K-H, Minkkinen I, Mäkinen MJ, Karttunen TJ, Tuomisto A (2019). Alterations in serum amino-acid profile in the progression of colorectal cancer: associations with systemic inflammation, tumour stage and patient survival. Br J Cancer.

[14] Geijsen A, Brezina S, Keski-Rahkonen P, Baierl A, Bachleitner-Hofmann T, Bergmann MM, Boehm J, Brenner H, Chang-Claude J, van Duijnhoven FJB (2019). Plasma metabolites associated with colorectal cancer: A discovery-replication strategy. Int J Cancer.

[15] Nishiumi S, Kobayashi T, Ikeda A, Yoshie T, Kibi M, Izumi Y (2012). A Novel Serum Metabolomics-Based Diagnostic Approach for Colorectal Cancer. PLoS One..

[16] Nishiumi S, Kobayashi T, Kawana S, Unno Y, Sakai T, Okamoto K, Yamada Y, Sudo K, Yamaji T, Saito Y (2017). Investigations in the possibility of early detection of colorectal cancer by gas chromatography/triple-quadrupole mass spectrometry. Oncotarget.

[17] Tan BB, Qiu YP, Zou X, Chen TL, Xie GX, Cheng Y, Dong TT, Zhao LJ, Feng B, Hu XF (2013). Metabonomics Identifies Serum Metabolite Markers of Colorectal Cancer. J Proteome Res.

[18] Erben V, Bhardwaj M, Schrotz-King P, Brenner H (2018). Metabolomics Biomarkers for Detection of Colorectal Neoplasms: A Systematic Review. Cancers.

[19] McCullough ML, Hodge RA, Campbell PT, Stevens VL, Wang Y (2021). Pre-Diagnostic Circulating Metabolites and Colorectal Cancer Risk in the Cancer Prevention Study-II Nutrition Cohort. Metabolites..

[20] Shu X, Xiang YB, Rothman N, Yu DX, Li HL, Yang G, Cai H, Ma X, Lan Q, Gao YT (2018). Prospective study of blood metabolites associated with colorectal cancer risk. Int J Cancer.

[21] Papadimitriou N, Gunter MJ, Murphy N, Gicquiau A, Achaintre D, Brezina S, Gumpenberger T, Baierl A, Ose J, Geijsen A (2021). Circulating tryptophan metabolites and risk of colon cancer: Results from case-control and prospective cohort studies. Int J Cancer.

[22] Riboli E, Hunt KJ, Slimani N, Ferrari P, Norat T, Fahey M, Charrondiere UR, Hemon B, Casagrande C, Vignat J (2002). European Prospective Investigation into Cancer and Nutrition (EPIC): study populations and data collection. Public Health Nutr..

[23] Riboli E, Kaaks R (1997). The EPIC Project: rationale and study design. European Prospective Investigation into Cancer and Nutrition. Int J Epidemiol.

[24] Allen N, Sudlow C, Downey P, Peakman T, Danesh J, Elliott P, Gallacher J, Green J, Matthews P, Pell J (2012). UK Biobank: Current status and what it means for epidemiology. Health Policy Technol.

[25] Romisch-Margl W, Prehn C, Bogumil R, Rohring C, Suhre K, Adamski J (2012). Procedure for tissue sample preparation and metabolite extraction for high-throughput targeted metabolomics. Metabolomics.

[26] Rothwell JA, Murphy N, Besevic J, Kliemann N, Jenab M, Ferrari P, Achaintre D, Gicquiau A, Vozar B, Scalbert A (2022). Metabolic Signatures of Healthy Lifestyle Patterns and Colorectal Cancer Risk in a European Cohort. Clin Gastroenterol Hepatol.

[27] Stepien M, Duarte-Salles T, Fedirko V, Floegel A, Barupal DK, Rinaldi S, Achaintre D, Assi N, Tjonneland A, Overvad K (2016). Alteration of amino acid and biogenic amine metabolism in hepatobiliary cancers: Findings from a prospective cohort study. Int J Cancer.

[28] Gu JP, Xiao YQ, Shu D, Liang XR, Hu XM, Xie YY (2019). Metabolomics Analysis in Serum from Patients with Colorectal Polyp and Colorectal Cancer by H-1-NMR Spectrometry. Dis Markers..

[29] Uchiyama K, Yagi N, Mizushima K, Higashimura Y, Hirai Y, Okayama T, Yoshida N, Katada K, Kamada K, Handa O (2017). Serum metabolomics analysis for early detection of colorectal cancer. J Gastroenterol.

[30] Li JK, Li J, Wang H, Qi LW, Zhu YM, Lai MD (2019). Tyrosine and Glutamine-Leucine Are Metabolic Markers of Early-Stage Colorectal Cancers. Gastroenterology.

[31] Kuhn T, Floegel A, Sookthai D, Johnson T, Rolle-Kampczyk U, Otto W, von Bergen M, Boeing H, Kaaks R (2016). Higher plasma levels of lysophosphatidylcholine 18:0 are related to a lower risk of common cancers in a prospective metabolomics study. BMC Med.

[32] Jutel M, Akdis M, Akdis CA (2009). Histamine, histamine receptors and their role in immune pathology. Clin Exp Allergy.

[33] Smolinska S, Jutel M, Crameri R, O'Mahony L (2014). Histamine and gut mucosal immune regulation. Allergy.

[34] Cianchi F, Cortesini C, Schiavone N, Perna F, Magnelli L, Fanti E, Bani D, Messerini L, Fabbroni V, Perigli G (2005). The role of cyclooxygenase-2 in mediating the effects of histamine on cell proliferation and vascular endothelial growth factor production in colorectal cancer. Clin Cancer Res.

[35] Feng RN, Niu YC, Sun XW, Li Q, Zhao C, Wang C, Guo FC, Sun CH, Li Y (2013). Histidine supplementation improves insulin resistance through suppressed inflammation in obese women with the metabolic syndrome: a randomised controlled trial. Diabetologia.

[36] Jobard E, Dossus L, Baglietto L, Fornili M, Lecuyer L, Mancini FR, Gunter MJ, Tredan O, Boutron-Ruault MC, Elena-Herrmann B (2021). Investigation of circulating metabolites associated with breast cancer risk by untargeted metabolomics: a case-control study nested within the French E3N cohort. Br J Cancer.

[37] Cluntun AA, Lukey MJ, Cerione RA, Locasale JW (2017). Glutamine Metabolism in Cancer: Understanding the Heterogeneity. Trends Cancer.

[38] Zhao YQ, Zhao X, Chen V, Feng Y, Wang L, Croniger C, Conlon RA, Markowitz S, Fearon E, Puchowicz M (2019). Colorectal cancers utilize glutamine as an anaplerotic substrate of the TCA cycle in vivo. Sci Rep.

[39] Ling HH, Pan Y-P, Fan C-W, Tseng W-K, Huang J-S, Wu T-H, Chou W-C, Wang C-H, Yeh K-Y, Chang P-H (2019). Clinical Significance of Serum Glutamine Level in Patients with Colorectal Cancer. Nutrients.

[40] Liu XR, Zheng Y, Guasch-Ferre M, Ruiz-Canela M, Toledo E, Clish C, Liang LM, Razquin C, Corella D, Estruch R (2019). High plasma glutamate and low glutamine-to-glutamate ratio are associated with type 2 diabetes: Case-cohort study within the PREDIMED trial. Nutr Metab Cardiovasc Dis.

[41] Morze J, Wittenbecher C, Schwingshackl L, Danielewicz A, Rynkiewicz A, Hu FB, Guasch-Ferre M (2022). Metabolomics and Type 2 Diabetes Risk: An Updated Systematic Review and Meta-analysis of Prospective Cohort Studies. Diabetes Care.

[42] Watson KM, Gaulke CA, Tsikitis VL (2020). Understanding the microbiome: a primer on the role of the microbiome in colorectal neoplasia. Ann Gastroenterol.

[43] Mardinoglu A, Shoaie S, Bergentall M, Ghaffari P, Zhang C, Larsson E, Bäckhed F, Nielsen J (2015). The gut microbiota modulates host amino acid and glutathione metabolism in mice. Mol Syst Biol.

[44] Salahshouri P, Emadi-Baygi M, Jalili M, Khan FM, Wolkenhauer O, Salehzadeh-Yazdi A (2021). A Metabolic Model of Intestinal Secretions: The Link between Human Microbiota and Colorectal Cancer Progression. Metabolites.

[45] Gao C, Major A, Rendon D, Lugo M, Jackson V, Shi Z, Mori-Akiyama Y, Versalovic J (2015). Histamine H2 Receptor-Mediated Suppression of Intestinal Inflammation by Probiotic Lactobacillus reuteri. MBIO.

[46] Pedersen HK, Gudmundsdottir V, Nielsen HB, Hyotylainen T, Nielsen T, Jensen BAH, Forslund K, Hildebrand F, Prifti E, Falony G (2016). Human gut microbes impact host serum metabolome and insulin sensitivity. Nature.

[47] Iguacel I, Schmidt JA, Perez-Cornago A, Van Puyvelde H, Travis R, Stepien M, Scalbert A, Casagrande C, Weiderpass E, Riboli E (2021). Associations between dietary amino acid intakes and blood concentration levels. Clin Nutr.

[48] Breier M, Wahl S, Prehn C, Fugmann M, Ferrari U, Weise M, Banning F, Seissler J, Grallert H, Adamski J (2014). Targeted Metabolomics Identifies Reliable and Stable Metabolites in Human Serum and Plasma Samples. PLoS One.

