# Supplementary material for: Circulating amino acid levels and colorectal cancer risk in the European Prospective Investigation into Cancer and Nutrition and UK Biobank cohorts
Source: BMC Med. 2023 Feb 28;21:80. doi: 10.1186/s12916-023-02739-4 (PMC9976469; doi:10.1186/s12916-023-02739-4)
Supplement: Supplementary file 1 — Additional file 1. Supplemental methods for laboratory analyses; Table S1, Reported coefficients of variation for 21 amino acids measured in the EPIC and UK Biobank cohorts; Table S2, Associations between 21 plasma or serum amino acids and colorectal, colon and rectal cancer risk in the EPIC and UK Biobank cohorts; Table S3, Associations between 21 plasma or serum amino acids and colorectal cancer risk in the EPIC and UK Biobank cohorts, by sex; Table S4, Associations between amino acids associated with colorectal cancer in either cohort in the main study, additionally adjusted for intakes of major sources of animal protein (red and processed meat, poultry, fish and dairy products) in the EPIC and UK Biobank cohorts; Table S5, Associations between concentrations of 21 plasma or serum amino acids and colorectal cancer risk in fasted participants only and all available participants in the EPIC nested case-control study. [file 12916_2023_2739_MOESM1_ESM.docx]

ADDITIONAL FILE 1

Rothwell et al.

## Circulating amino acid levels and colorectal cancer risk in the European Prospective Investigation into Cancer and Nutrition and UK Biobank cohorts

# Supplemental Methods

### Sample preparation and amino acid measurement in EPIC

Serum and plasma samples were stored at the International Agency for Research on Cancer (IARC), Lyon, France at -196°C in liquid nitrogen, apart from those of Sweden (-80°C freezers) and Denmark (-150°C nitrogen vapor). Data and biospecimens used were from all EPIC countries except Greece.

Targeted metabolomics using the Biocrates platform was performed at IARC or the Helmholtz Zentrum, München, Germany (all other case-control samples). The Absolute*IDQ*^TM^ p150 or p180 Kits (BIOCRATES Life Sciences AG, Innsbruck, Austria) were used to measure concentrations of amino acids, biogenic amines, hexose sugars, acylcarnitines, sphingolipids (SM), phosphatidylcholines (PC) and lysophosphatidylcholines (lysoPC) in serum or plasma, following the recommended procedure.

The analysis conducted at IARC employed a 1290 Series liquid chromatography (LC) instrument with a Q-Trap 5500 mass spectrometer (Agilent Technologies, Les Ulis, France). The Helmholtz method was based on a 1200 Series LC instrument (Agilent, Böblingen, Germany) with an API 4000 triple quadrupole mass spectrometer (AB Sciex, Darmstadt, Germany). Case-control pairs were analyzed in the same batch, and coefficients of variation (CV) were calculated for each metabolite. Full details of laboratory procedures have been published. Amino acids were fully quantified in μmol/L.

**Sample preparation and amino acid measurement in the UK Biobank**

The prepared samples were loaded onto a cooled sample changer, which maintains the temperature of samples waiting to be measured at +6°C. Two NMR spectra were recorded for each plasma sample using a 500 MHz NMR spectrometer (Bruker AVANCE IIIHD, Bruker, Coventry, UK). The first spectrum is a pre-saturated proton NMR spectrum, which features resonances arising mainly from proteins and lipids within various lipoprotein particles. The other spectrum is a T2-relaxation-filtered spectrum where most of the broad macromolecule and lipoprotein lipid signals are suppressed, leading to enhanced detection of low-molecular-weight metabolites. Automated quality control of the spectral data was performed. The metabolic biomarkers were quantified using Nightingale Health’s proprietary software (Nightingale Health biomarker quantification library 2020).

# Supplemental Tables and Figures

**Table S1. Reported coefficients of variation for 21 amino acids measured in the EPIC and UK Biobank cohorts.**

| Amino acid | CV EPIC laboratory 1^a^ | CV EPIC laboratory 2^a^ | CV UK Biobank^b^ |
| --- | --- | --- | --- |
| Alanine | 6.3 | - | < 5 |
| Arginine | 5.2 | 8.1 | - |
| Asparagine | 6.4 | - | - |
| Aspartate | 11.5 | - | - |
| Citrulline | 7.2 | - | - |
| Glutamine | 7.6 | 8 | < 5 |
| Glutamate | 5.7 | - | - |
| Glycine | 6.9 | 7.3 | < 5 |
| Histidine | 4.5 | 7.5 | < 5 |
| Isoleucine | 7.1 | - | < 5 |
| Leucine | 6.9 | - | < 5 |
| Lysine | 9.4 | - | - |
| Methionine | 11.4 | 9.5 | - |
| Ornithine | 11.6 | 7.2 | - |
| Phenylalanine | 6.2 | 8 | < 5 |
| Proline | 5 | 6.8 | - |
| Serine | 5 | 7.3 | - |
| Threonine | 6.1 | 7.3 | - |
| Tryptophan | 8 | 7.1 | - |
| Tyrosine | 6.5 | 8.3 | < 5 |
| Valine | 9.1 | 6.9 | < 5 |

CV, coefficient of variation; NA, not available.

^a^Laboratory 1: International Agency for Research on Cancer; 13 plates of serum samples with two QCs per plate for endogenous compounds, 56 batches of plasma samples, two QCs per batch for fatty acids. Laboratory 2: Helmholtz Zentrum; 29 plates of serum samples with 5 aliquots of a reference serum as a QC.

^b^Nightingale Health metabolic biomarker profiling platform.

**Table S2. Associations between 21 plasma or serum amino acids and colorectal, colon, and rectal cancer risk in the EPIC and UK Biobank cohorts.**

| Amino acid | Cohort | Colorectal cancer |  |  | Colon cancer |  | Rectal cancer |  |  |
| --- | --- | --- | --- | --- | --- | --- | --- | --- | --- |
|  |  | OR/HR per SD increase in concentration (95% CI) ^a^ | Cases | FDR *P*-value | OR/HR per SD increase in concentration (95% CI) ^a^ | Cases | OR/HR per SD increase in concentration (95% CI) ^a^ | Cases | *P*-heterogeneity, colon versus rectal cancer |
| **Measured in EPIC and the UK Biobank cohorts** |  |  |  |  |  |  |  |  |  |
| Glycine | EPIC | 0.89 (0.75-1.05) | 654 | 0.45 | 0.91 (0.76-1.08) | 625 | - | 29 | - |
|  | UK Biobank | 1.03 (0.96-1.10) | 1221 | 0.68 | 1.04 (0.97-1.13) | 833 | 0.99 (0.88-1.13) | 388 | 0.51 |
|  |  |  |  |  |  |  |  |  |  |
| Glutamine | EPIC | **0.85 (0.75-0.97)** | 654 | 0.08 | **0.85 (0.75-0.97)** | 625 | - | 29 | - |
|  | UK Biobank | 0.95 (0.89-1.01) | 1221 | 0.39 | **0.92 (0.85-0.99)** | 833 | 1.02 (0.91-1.13) | 388 | 0.13 |
|  |  |  |  |  |  |  |  |  |  |
| Histidine | EPIC | **0.80 (0.69-0.92)** | 654 | 0.03 | **0.78 (0.67-0.90)** | 625 | - | 29 | - |
|  | UK Biobank | **0.93 (0.87-0.99)** | 1221 | 0.17 | 0.95 (0.88-1.02) | 833 | **0.89 (0.80-0.99)** | 388 | 0.37 |
|  |  |  |  |  |  |  |  |  |  |
| Alanine | EPIC (p180) | 1.04 (0.89-1.22) | 354 | 0.75 | 1.03 (0.88-1.21) | 350 | **-** | 4 | - |
|  | UK Biobank | 1.03 (0.96-1.10) | 1221 | 0.68 | 1.03 (0.95-1.12) | 833 | 1.01 (0.90-1.14) | 388 | 0.78 |
|  |  |  |  |  |  |  |  |  |  |
| Phenylalanine | EPIC (p180) | 0.89 (0.77-1.03) | 354 | 0.45 | 0.89 (0.77-1.04) | 350 | - | 4 | - |
|  | UK Biobank | 0.99 (0.93-1.05) | 1221 | 0.82 | 1.02 (0.95-1.10) | 833 | 0.92 (0.82-1.03) | 388 | 0.09 |
|  |  |  |  |  |  |  |  |  |  |
| Tyrosine | EPIC | 0.90 (0.76-1.06) | 654 | 0.45 | 0.88 (0.74-1.04) | 625 | - | 29 | - |
|  | UK Biobank | 0.99 (0.93-1.05) | 1221 | 0.82 | 0.99 (0.92-1.06) | 833 | 0.98 (0.88-1.09) | 388 | 0.92 |
|  |  |  |  |  |  |  |  |  |  |
| Valine | EPIC | 0.92 (0.78-1.08) | 654 | 0.47 | 0.91 (0.77-1.08) | 625 | - | 29 | - |
|  | UK Biobank | 0.97 (0.91-1.04) | 1221 | 0.68 | 0.96 (0.89-1.04) | 833 | 1.00 (0.89-1.11) | 388 | 0.62 |
|  |  |  |  |  |  |  |  |  |  |
| Isoleucine | EPIC (p180) | 0.92 (0.75-1.14) | 354 | 0.64 | 0.90 (0.73-1.11) | 350 | - | 4 | - |
|  | UK Biobank | 0.99 (0.94-1.05) | 1221 | 0.82 | 0.97 (0.91-1.05) | 833 | 1.03 (0.93-1.14) | 388 | 0.38 |
|  |  |  |  |  |  |  |  |  |  |
| Leucine | EPIC (p180) | **0.79 (0.64-0.98)** | 354 | 0.15 | **0.78 (0.63-0.96)** | 350 | - | 4 | - |
|  | UK Biobank | 0.96 (0.90-1.02) | 1221 | 0.46 | 0.93 (0.86-1.00) | 833 | 1.01 (0.91-1.12) | 388 | 0.23 |
| **Measured In EPIC only** |  |  |  |  |  |  |  |  |  |
| Aspartate | EPIC (p180) | 0.99 (0.81-1.21) | 354 | 0.91 | 1.00 (0.81-1.22) | 350 | - | 4 | - |
| Glutamate | EPIC (p180) | 1.10 (0.85-1.43) | 354 | 0.64 | 1.11 (0.85-1.44) | 350 | - | 4 | - |
| Serine | EPIC | 0.89 (0.73-1.08) | 654 | 0.45 | 0.87 (0.71-1.06) | 625 | - | 29 | - |
| Arginine | EPIC | 1.02 (0.83-1.24) | 654 | 0.87 | 0.99 (0.81-1.22) | 625 | - | 29 | - |
| Methionine | EPIC | 0.88 (0.69-1.13) | 654 | 0.47 | 0.86 (0.67-1.12) | 625 | - | 29 | - |
| Tryptophan | EPIC | 0.85 (0.64-1.12) | 654 | 0.45 | 0.84 (0.63-1.11) | 625 | - | 29 | - |
| Proline | EPIC | 1.06 (0.93-1.20) | 654 | 0.48 | 1.07 (0.94-1.21) | 625 | - | 29 | - |
| Lysine | EPIC (p180) | **0.78 (0.66-0.93)** | 354 | 0.05 | **0.78 (0.65-0.92)** | 350 | - | 4 | - |
| Ornithine | EPIC | 0.88 (0.69-1.13) | 654 | 0.47 | 1.02 (0.85-1.21) | 625 | - | 29 | - |
| Citrulline | EPIC (p180) | 1.15 (0.97-1.37) | 354 | 0.40 | 1.17 (0.98-1.39) | 350 | - | 4 | - |
| Asparagine | EPIC (p180) | 1.08 (0.91-1.28) | 354 | 0.58 | 1.07 (0.90-1.27) | 350 | - | 4 | - |
| Threonine | EPIC | 0.96 (0.85-1.09) | 654 | 0.60 | 0.95 (0.84-1.08) | 625 | - | 29 | - |

OR, odds ratio; HR, hazard ratio; FDR, false discovery rate. Estimates for which 95% CI do not include 1 are given in bold text.

^a^ Multivariable models were adjusted for smoking status (never, former, and current smoker), alcohol use (never, former, only at recruitment, and lifetime drinker), physical activity at recruitment (inactive, moderately inactive, moderately active, active) and body mass index (<25, 25-30 and >30 kg/m^2^). In UK Biobank, categories differed slightly for total physical activity (<10, 10-20, 20-40, 40-60, >60 metabolic equivalent of task [MET] hours/week), alcohol consumption frequency (never, special occasions only, 1–3 times/month, 1–2 times per week, 3–4 times/week, daily or almost daily, unknown/prefer not to answer), and were also adjusted for family history of colorectal cancer (yes/no) and time since last meal (hours).

**Table S3. Associations between 21 plasma or serum amino acids and colorectal cancer risk in the EPIC and UK Biobank cohorts, by sex.**

| Amino acid | Cohort | OR/HR per SD increase in concentration (95% CI) ^a^ | | | *P*-heterogeneity, female versus male |
| --- | --- | --- | --- | --- | --- |
|  |  | All participants | Women | Men |  |
| **Measured in EPIC and the UK Biobank cohorts** |  |  |  |  |  |
| Glycine | EPIC | 0.89 (0.75-1.05) | 0.91 (0.73-1.14) | 0.80 (0.60-1.05) | 0.36 |
|  | UK Biobank | 1.03 (0.96-1.10) | 1.03 (0.95-1.11) | 1.02 (0.91-1.14) | 0.33 |
|  |  |  |  |  |  |
| Glutamine | EPIC | **0.85 (0.75-0.97)** | 0.89 (0.76-1.05) | **0.80 (0.66-0.97)** | 0.44 |
|  | UK Biobank | 0.95 (0.89-1.01) | 0.96 (0.87-1.05) | 0.94 (0.87-1.02) | 0.52 |
|  |  |  |  |  |  |
| Histidine | EPIC | **0.80 (0.69-0.92)** | 0.84 (0.69-1.01) | **0.75 (0.60-0.94)** | 0.54 |
|  | UK Biobank | **0.93 (0.87-0.99)** | 1.00 (0.91-1.10) | **0.88 (0.81-0.95)** | 0.02 |
|  |  |  |  |  |  |
| Alanine | EPIC (p180) | 1.04 (0.89-1.22) | 1.14 (0.92-1.41) | 0.95 (0.74-1.21) | 0.44 |
|  | UK Biobank | 1.03 (0.96-1.10) | 0.98 (0.88-1.09) | 1.06 (0.97-1.16) | 0.50 |
|  |  |  |  |  |  |
| Phenylalanine | EPIC (p180) | 0.89 (0.77-1.03) | 1.02 (0.83-1.26) | **0.77 (0.63-0.95)** | 0.17 |
|  | UK Biobank | 0.99 (0.93-1.05) | 1.03 (0.94-1.13) | 0.96 (0.88-1.04) | 0.29 |
|  |  |  |  |  |  |
| Tyrosine | EPIC | 0.90 (0.76-1.06) | 0.93 (0.75-1.16) | 0.86 (0.67-1.11) | 0.97 |
|  | UK Biobank | 0.99 (0.93-1.05) | 0.99 (0.91-1.08) | 0.98 (0.91-1.06) | 0.61 |
|  |  |  |  |  |  |
| Valine | EPIC | 0.92 (0.78-1.08) | 1.01 (0.82-1.24) | 0.83 (0.65-1.05) | 0.80 |
|  | UK Biobank | 0.97 (0.91-1.04) | 1.01 (0.92-1.11) | 0.95 (0.87-1.03) | 0.51 |
|  |  |  |  |  |  |
| Isoleucine | EPIC (p180) | 0.92 (0.75-1.14) | 1.09 (0.86-1.37) | 0.74 (0.53-1.01) | 0.24 |
|  | UK Biobank | 0.99 (0.94-1.05) | 1.04 (0.96-1.14) | 0.96 (0.88-1.03) | 0.25 |
|  |  |  |  |  |  |
| Leucine | EPIC (p180) | **0.79 (0.64-0.98)** | 0.92 (0.72-1.17) | **0.68 (0.50-0.93)** | 0.38 |
|  | UK Biobank | 0.96 (0.90-1.02) | 0.99 (0.90-1.09) | 0.93 (0.86-1.01) | 0.51 |
| **Measured In EPIC only** |  |  |  |  |  |
| Aspartate | EPIC (p180) | 0.99 (0.81-1.21) | 0.94 (0.72-1.24) | 1.04 (0.71-1.51) | 0.55 |
| Glutamate | EPIC (p180) | 1.10 (0.85-1.43) | 1.01 (0.74-1.37) | 1.23 (0.76-1.97) | 0.40 |
| Serine | EPIC | 0.89 (0.73-1.08) | 0.96 (0.74-1.25) | 0.80 (0.60-1.08) | 0.45 |
| Arginine | EPIC | 1.02 (0.83-1.24) | 1.22 (0.93-1.60) | 0.85 (0.63-1.15) | 0.24 |
| Methionine | EPIC | 0.88 (0.69-1.13) | 0.99 (0.71-1.39) | 0.79 (0.55-1.15) | 0.64 |
| Tryptophan | EPIC | 0.85 (0.64-1.12) | 0.97 (0.66-1.41) | 0.72 (0.46-1.11) | 0.68 |
| Proline | EPIC | 1.06 (0.93-1.20) | 1.01 (0.86-1.19) | 1.14 (0.95-1.38) | 0.35 |
| Lysine | EPIC (p180) | **0.78 (0.66-0.93)** | **0.77 (0.62-0.96)** | 0.80 (0.61-1.04) | 0.76 |
| Ornithine | EPIC | 0.88 (0.69-1.13) | 1.04 (0.82-1.32) | 0.99 (0.78-1.26) | 0.91 |
| Citrulline | EPIC (p180) | 1.15 (0.97-1.37) | **1.34 (1.06-1.70)** | 0.95 (0.72-1.24) | 0.08 |
| Asparagine | EPIC (p180) | 1.08 (0.91-1.28) | 1.18 (0.94-1.47) | 1.01 (0.77-1.32) | 0.30 |
| Threonine | EPIC | 0.96 (0.85-1.09) | 1.03 (0.87-1.21) | 0.89 (0.73-1.08) | 0.19 |

OR, odds ratio; HR, hazard ratio; FDR, false discovery rate; Q, quartile. Estimates for which 95% CI do not include 1 are given in bold text.

^a^ Multivariable models were adjusted for smoking status (never, former, and current smoker), alcohol use (never, former, only at recruitment, and lifetime drinker), physical activity at recruitment (inactive, moderately inactive, moderately active, active) and body mass index (<25, 25-30 and >30 kg/m^2^). In UK Biobank, categories differed slightly for total physical activity (<10, 10-20, 20-40, 40-60, >60 metabolic equivalent of task [MET] hours/week), alcohol consumption frequency (never, special occasions only, 1–3 times/month, 1–2 times per week, 3–4 times/week, daily or almost daily, unknown/prefer not to answer), and were also adjusted for family history of colorectal cancer (yes/no) and time since last meal (hours).

**Table S4. Associations between amino acids associated with colorectal cancer in either cohort in the main study, additionally adjusted for intakes of major sources of animal protein (red and processed meat, poultry, fish, and dairy products) in the EPIC and UK Biobank cohorts.**

| Amino acid | Cohort | Base co-variates only ^a^ | | Adjusted for major sources of animal proteins ^b^ | |
| --- | --- | --- | --- | --- | --- |
|  |  | OR/HR per SD increase in concentration (95% CI) | FDR *P*-value | OR/HR per SD increase in concentration (95% CI) | FDR *P*-value |
| **Measured in EPIC and the UK Biobank cohorts** |  |  |  |  |  |
| Glycine | EPIC | 0.89 (0.75-1.05) | 0.45 | 0.87 (0.73-1.04) | 0.49 |
|  | UK Biobank | 1.03 (0.96-1.10) | 0.68 | 1.03 (0.96-1.10) | 0.81 |
|  |  |  |  |  |  |
| Glutamine | EPIC | **0.85 (0.75-0.97)** | 0.08 | **0.84 (0.74-0.95)** | 0.05 |
|  | UK Biobank | 0.95 (0.89-1.01) | 0.39 | 0.95 (0.89-1.01) | 0.41 |
|  |  |  |  |  |  |
| Histidine | EPIC | **0.80 (0.69-0.92)** | 0.03 | **0.79 (0.68-0.91)** | 0.04 |
|  | UK Biobank | **0.93 (0.87-0.99)** | 0.17 | 0.93 (0.87-0.99) | 0.24 |
|  |  |  |  |  |  |
| Alanine | EPIC (p180) | 1.04 (0.89-1.22) | 0.75 | 1.03 (0.87-1.21) | 0.88 |
|  | UK Biobank | 1.03 (0.96-1.10) | 0.68 | 1.03 (0.96-1.10) | 0.81 |
|  |  |  |  |  |  |
| Phenylalanine | EPIC (p180) | 0.89 (0.77-1.03) | 0.45 | 0.89 (0.77-1.03) | 0.49 |
|  | UK Biobank | 0.99 (0.93-1.05) | 0.82 | 0.99 (0.94-1.06) | 0.97 |
|  |  |  |  |  |  |
| Tyrosine | EPIC | 0.90 (0.76-1.06) | 0.45 | 0.90 (0.76-1.06) | 0.50 |
|  | UK Biobank | 0.99 (0.93-1.05) | 0.82 | 1.00 (0.94-1.06) | 0.97 |
|  |  |  |  |  |  |
| Valine | EPIC | 0.92 (0.78-1.08) | 0.47 | 0.91 (0.77-1.07) | 0.52 |
|  | UK Biobank | 0.97 (0.91-1.04) | 0.68 | 0.98 (0.92-1.05) | 0.85 |
|  |  |  |  |  |  |
| Isoleucine | EPIC (p180) | 0.92 (0.75-1.14) | 0.64 | 0.90 (0.73-1.11) | 0.53 |
|  | UK Biobank | 0.99 (0.94-1.05) | 0.82 | 1.00 (0.94-1.06) | 0.97 |
|  |  |  |  |  |  |
| Leucine | EPIC (p180) | **0.79 (0.64-0.98)** | 0.15 | **0.78 (0.65-0.92)** | 0.12 |
|  | UK Biobank | 0.96 (0.90-1.02) | 0.46 | 0.97 (0.91-1.03) | 0.81 |
| **Measured In EPIC only** |  |  |  |  |  |
| Aspartate | EPIC (p180) | 0.99 (0.81-1.21) | 0.91 | 0.98 (0.80-1.20) | 0.88 |
| Glutamate | EPIC (p180) | 1.10 (0.85-1.43) | 0.64 | 1.12 (0.86-1.47) | 0.58 |
| Serine | EPIC | 0.89 (0.73-1.08) | 0.45 | 0.88 (0.72-1.07) | 0.50 |
| Arginine | EPIC | 1.02 (0.83-1.24) | 0.87 | 1.02 (0.83-1.25) | 0.88 |
| Methionine | EPIC | 0.88 (0.69-1.13) | 0.47 | 0.87 (0.67-1.12) | 0.52 |
| Tryptophan | EPIC | 0.85 (0.64-1.12) | 0.45 | 0.86 (0.64-1.14) | 0.52 |
| Proline | EPIC | 1.06 (0.93-1.20) | 0.48 | 1.04 (0.91-1.18) | 0.71 |
| Lysine | EPIC (p180) | **0.78 (0.66-0.93)** | 0.05 | **0.78 (0.65-0.92)** | 0.05 |
| Ornithine | EPIC | 0.88 (0.69-1.13) | 0.47 | 1.01 (0.85-1.21) | 0.88 |
| Citrulline | EPIC (p180) | 1.15 (0.97-1.37) | 0.40 | 1.14 (0.95-1.35) | 0.49 |
| Asparagine | EPIC (p180) | 1.08 (0.91-1.28) | 0.58 | 1.09 (0.92-1.30) | 0.52 |
| Threonine | EPIC | 0.96 (0.85-1.09) | 0.60 | 0.95 (0.84-1.08) | 0.62 |

OR, odds ratio; HR, hazard ratio; FDR, false discovery rate; Q, quartile. Estimates for which 95% CI do not include 1 are given in bold text.

^a^ Multivariable models were adjusted for smoking status (never, former, and current smoker), alcohol use (never, former, only at recruitment, and lifetime drinker), physical activity at recruitment (inactive, moderately inactive, moderately active, active) and body mass index (<25, 25-30 and >30 kg/m^2^). In UK Biobank, categories differed slightly for total physical activity (<10, 10-20, 20-40, 40-60, >60 metabolic equivalent of task [MET] hours/week), alcohol consumption frequency (never, special occasions only, 1–3 times/month, 1–2 times per week, 3–4 times/week, daily or almost daily, unknown/prefer not to answer), and were also adjusted for family history of colorectal cancer (yes/no) and time since last meal (hours).

^b^ Assessed by food frequency questionnaire data in grams/day.

**Table S5. Associations between concentrations of 21 plasma or serum amino acids and colorectal cancer risk in fasted participants only and all available participants in the EPIC nested case-control study.**

|  | Fasting subset only (main study) | | | All available participants | | |
| --- | --- | --- | --- | --- | --- | --- |
| Amino acid (by decreasing blood concentration) | Colorectal cancer cases ^a^ | OR per SD concentration (95% CI) ^b^ | FDR *P*-value | Colorectal cancer cases ^a^ | OR per SD concentration (95% CI) ^b^ | FDR P-value |
| Glutamine | 654 | **0.85 (0.75-0.97)** | 0.08 | 3216 | **0.90 (0.84-0.98)** | 0.08 |
| Alanine | 354 | 1.04 (0.89-1.22) | 0.75 | 934 | 1.01 (0.88-1.16) | 0.98 |
| Glycine | 654 | 0.89 (0.75-1.05) | 0.45 | 3216 | 0.94 (0.84-1.04) | 0.70 |
| Valine | 654 | 0.92 (0.78-1.08) | 0.47 | 3216 | 0.99 (0.90-1.09) | 0.98 |
| Lysine | 354 | **0.78 (0.66-0.93)** | 0.05 | 934 | **0.80 (0.69-0.93)** | 0.08 |
| Proline | 654 | 1.06 (0.93-1.20) | 0.48 | 3216 | 1.02 (0.94-1.10) | 0.90 |
| Serine | 654 | 0.89 (0.73-1.08) | 0.45 | 3216 | 0.95 (0.84-1.07) | 0.73 |
| Leucine | 354 | **0.79 (0.64-0.98)** | 0.15 | 934 | 0.89 (0.75-1.05) | 0.70 |
| Threonine | 654 | 0.96 (0.85-1.09) | 0.60 | 3216 | 0.97 (0.89-1.05) | 0.73 |
| Ornithine | 654 | 0.88 (0.69-1.13) | 0.47 | 3216 | 0.98 (0.87-1.11) | 0.90 |
| Arginine | 654 | 1.02 (0.83-1.24) | 0.87 | 3216 | 1.07 (0.95-1.21) | 0.70 |
| Tyrosine | 654 | 0.90 (0.76-1.06) | 0.45 | 3216 | 0.96 (0.88-1.05) | 0.73 |
| Histidine | 654 | **0.80 (0.69-0.92)** | 0.03 | 3216 | **0.87 (0.78-0.97)** | 0.08 |
| Tryptophan | 654 | 0.85 (0.64-1.12) | 0.45 | 3216 | 0.95 (0.82-1.10) | 0.84 |
| Isoleucine | 354 | 0.92 (0.75-1.14) | 0.64 | 934 | 1.00 (0.84-1.20) | 0.98 |
| Phenylalanine | 354 | 0.89 (0.77-1.03) | 0.45 | 3216 | 0.94 (0.85-1.03) | 0.70 |
| Glutamate | 354 | 1.10 (0.85-1.43) | 0.64 | 934 | 1.13 (0.90-1.43) | 0.70 |
| Asparagine | 354 | 1.08 (0.91-1.28) | 0.58 | 934 | 0.96 (0.80-1.16) | 0.90 |
| Methionine | 654 | 0.88 (0.69-1.13) | 0.47 | 3216 | 0.98 (0.87-1.10) | 0.90 |
| Citrulline | 354 | 1.15 (0.97-1.37) | 0.40 | 934 | 1.16 (1.00-1.36) | 0.30 |
| Aspartate | 354 | 0.99 (0.81-1.21) | 0.91 | 934 | 0.96 (0.80-1.16) | 0.90 |

OR, odds ratio; FDR, false discovery rate. Estimates for which 95% CI do not include 1 are given in bold text.

^a^ Amino acids measured using the Biocrates AbsoluteIDQ^TM^ p180 kit were measured in a subset of the available participants only.

^b^ Multivariable models were adjusted for smoking status (never, former, and current smoker), alcohol use (never, former, only at recruitment, and lifetime drinker), physical activity at recruitment (inactive, moderately inactive, moderately active, active) and body mass index (<25, 25-30 and >30 kg/m^2^).
